# Impact of Sex and Obesity on Echocardiographic Parameters in Children and Adolescents

**DOI:** 10.1007/s00246-022-02876-2

**Published:** 2022-04-08

**Authors:** Jeannine von der Born, Sarah Baberowski, Nima Memaran, Lena Grams, Denise Homeyer, Bianca Borchert-Mörlins, Rizky Indrameikha Sugianto, Mira Paulsen, Elena Bauer, Carl Grabitz, Bernhard M. W. Schmidt, Arno Kerling, Philipp Beerbaum, Meike Stiesch, Uwe Tegtbur, Anette Melk

**Affiliations:** 1 Department of Pediatric Kidney, Liver and Metabolic Diseases, Hannover Medical School, Carl-Neuberg-Str. 1, 30625 Hannover, Germany; 2 Institute of Sports Medicine, Hannover Medical School, Hannover, Germany; 3 Department of Cardiology and Angiology, Hannover Medical School, Hannover, Germany; 4 Department of Prosthetic Dentistry and Biomedical Material Research, Hannover Medical School, Hannover, Germany; 5 Department of Nephrology and Hypertension, Hannover Medical School, Hannover, Germany; 6 Department of Pediatric Cardiology and Pediatric Intensive Care Medicine, Hannover Medical School, Hannover, Germany

**Keywords:** Children, Obesity, Echocardiography, Sex differences, Reference values

## Abstract

Subclinical alterations in left ventricular structure and function are detectable in adolescents with hypertension or obesity. However, data on early echocardiographic abnormalities in seemingly healthy children are lacking. Sex differences in cardiac structure and function have been previously reported, but sex-specific reference values are not available. Specifically, the potential interaction of sex and overweight has not been addressed at all. Anthropometric data, blood pressure and exercise tests were obtained in 356 healthy children. Echocardiographic parameters comprised peak early (*E*) and late (*A*) mitral inflow Doppler velocities, *E*/*A *ratio, tissue Doppler peak velocities of early (*e*′) and late diastolic (*a*′) excursion of mitral/septal annulus and isovolumetric relaxation time (IVRT). Left ventricular mass index (LVMI) and LVMI *z*-score were calculated. Interaction terms between BMI and sex and stratification by sex were used for analysis. We provide values for echocardiographic parameters for children of two age groups separated by BMI. Overweight/obese children had a significant higher LVMI, lower *E*/*A *ratio, higher *E*/*e*′ ratios and a longer IVRT. For a given BMI in the upper ranges we demonstrated a higher LVMI in girls than in boys, the IVRT extended significantly more in girls than in boys with increasing BMI. There are sex differences in structural and functional echocardiographic parameters in children and adolescents. Our data not only confirms the importance of overweight and obesity, but demonstrates important interactions between sex and overweight. The greater susceptibility of overweight girls toward echocardiographic changes associated with potential long-term functional impairment needs further exploration and follow-up.

Trial registration number DRKS00012371; Date 18.08.2017.

**Supplementary Information:**

The online version contains supplementary material available at 10.1007/s00246-022-02876-2.

## Introduction

With the recognition of obesity as an important determinant of cardiac damage and remodeling, the early diagnosis of cardiac alterations in children is becoming more important especially in conjunction with the rise of childhood obesity. Early changes in left ventricular structure and function are already detected in adolescents with cardiovascular risk factors, like hypertension or adiposity [1]. Elevated LVMI [2], as well as indices of a lower ventricular compliance in overweight and obese children compared to their lean counterparts were described [3, 4]. Excessive obesity was linked to diastolic dysfunction in children, independent of comorbidities [5].

While the existing data suggest that these changes start very early in life, there is a lack of substantial data on early changes of echocardiographic parameters in children with obesity. The effect of sex has not been addressed to date; sex-specific reference values are not available. In light of different sex-specific phenotypes of cardiac disease in adulthood [6] differences between the sexes in childhood concerning cardiac function and structure should be taken into account. A recent paper investigated children with chronic kidney disease as a high risk group for cardiovascular comorbidities and demonstrated a higher susceptibility for girls toward higher LVMI especially when they were overweight [7]. While, in general, female sex is believed to be cardioprotective numerous studies have shown that in presence of special risk factors women become more susceptible. The situation in childhood, especially prior to puberty as well as a sex-specific influence of BMI on echocardiographic parameters has not been addressed at all.

The aim of our study was to describe structural and functional echocardiographic parameters in children and adolescents of two age groups separated by sex and BMI. Based on our data, we were able to characterize influencing factors associated with the measured parameters and can show that sex-specific differences occur in presence of the important CV risk factor overweight and obesity.

## Methods

### Study Design and Cohort

Three primary and two secondary schools were randomly chosen. All pupils from the 2nd grade, or 5th grade, respectively, were invited to participate in this cross-sectional prospective study from April to July 2017. Exclusion criteria included structural or congenital heart disease. All parents and children gave written informed consent before participation. The study was approved by the Institutional Review Board (No. 7290) and performed according to the Declaration of Helsinki.

### Anthropometric, Blood Pressure and Physical Fitness Assessment

Height, weight and waist circumference were measured, waist-circumference *z*-score was calculated according to Sharma et al. [8]. Body surface area (BSA) was calculated according to Du Bois [9]. Body mass index *z*-score (BMIz) was calculated using WHO reference growth standards [10]. We separated our study group according to children’s BMIz in a group with a BMIz < 1.04 and in an overweight/obese group with a BMIz ≥ 1.04. Age was classified into a group of 7–9 and 10–13 year olds, enabling the comparison of echocardiographic parameters to the published reference values from Eidem et al. [11]. Blood pressure (BP) measurement was performed in accordance with current guidelines [12, 13]. In short, BP was measured in a seated position after 5 min of rest, three times on both arms, with at least a 2-min interval between measurements. We used the oscillometric Dinamap device (Dinamap v150; Fa. GE Medical Systems, Chicago, Illinois, USA). The mean was calculated from the second and third measurements, and normalized for sex, age, and height, expressed as *z*-score [12, 13]. Physical fitness was assessed by graded exercise tests on an ergometer bicycle (sitting position) according to a modified Godfrey protocol [14]. The workload was increased stepwise by 15 W at 1-min intervals. All subjects were encouraged to exercise until exhaustion (breathlessness and leg muscle pain and/or a heart rate ≥ 85% of maximum (calculated 220—age in years) [15].

### Echocardiographic Measurements

Transthoracic echocardiography was performed by two uniformly trained investigators. Both were specialized on pediatric echocardiography and followed a standardized protocol using a PHILIPS CX 50 ultrasound machine (Philips Medical Systems, Bothell, USA) equipped with a 5 MHz transducer. Inter- and intra-observer variability was within the range formerly reported by Colan et al. [16]. A segmental analysis was performed to assure segmental anatomy and to exclude a congenital heart defect in accordance with the Guidelines of the American Society of Echocardiography [17].

Left and right ventricular end-diastolic wall thickness and end-diastolic dimensions were obtained from the parasternal short axis view at the level of the papillary muscles using M-mode. LVMI was defined as LV mass/*ht*^2.16^ as proposed by Chinali et al. [18]. LVM *z*-score (LVMz) according to height was calculated as described by Foster et al. [19], LVMIz-for-lean body mass (LBM) was calculated as proposed by Foster et al. [20], both methods of normalization were used for further analysis. Left ventricular end-diastolic dimension *z*-score (LVEDdz) was calculated according to Lopez et al. [21]. Ejection fraction was measured by the biplane modified Simpson method. For diastolic function pulsed Doppler measurements of mitral and tricuspid inflow (E-, A-wave) were performed in the apical four-chamber view, with the sample volume positioned between the tips of the mitral leaflets within ± 15° of the central volume stream, the *E*/*A *ratio was calculated. The time intervals of isovolumetric relaxation time (IVRT) and isovolumetric contraction time (IVCT) were measured from the apical five chamber view using pulse-wave (PW) Doppler positioned to record left ventricular inflow and outflow tract simultaneously. To determine the IVRT, the time interval from the ending of the aortic flow to the beginning of the mitral inflow was measured. For the determination of IVCT, the time interval from the ending of the atrioventricular valve inflow to the beginning of the ventricular outflow was measured. For the further assessment of diastolic function the following PW Tissue Doppler parameters were obtained: peak velocities of early (*e*′) and late diastolic (*a*′) excursion of the lateral and septal mitral annulus as well as of the tricuspid annulus. IVRT, obtained by tissue Doppler, was measured from the end of the S-wave to beginning of the following e-wave. Pulmonary venous inflow (PV) was measured in an apical four-chamber view, with the PW sample volume placed as far as possible in the right upper pulmonary vein. The recordings comprised peak systolic velocity (S-wave) as well as peak diastolic velocity (D-wave). Five consecutive cardiac cycles were recorded from every approach. All parameters were measured five times, the median value was taken for further analysis. All measurements were performed by a single investigator. For any given structure, measurements were made only if excellent and unambiguous views were available. Thus, not all structures were measured in all patients.

### Statistical Analysis

Our endpoints comprised different echocardiographic parameters (LVMI, LVMIz, mitral *E*/*A* ratio, mitral annular *E*/*e*′, septal annular *E*/*e*′, septal annular IVRT, tricuspid *E*/*A* ratio). Data are given as mean ± standard deviations (SD) or absolute and relative frequencies. Echocardiographic parameters were compared between age, sex and BMI categories. Differences in echocardiographic parameters between boys and girls were assessed using *t* test. Potential covariates were selected for each outcome variable based on prior knowledge. Backward linear regression modeling with a *p* value of 0.2 or less as selection criteria were performed. Linear regression models for LVMI, LVMz, LVMIz, septal annular IVRT and PV flow D-wave velocity including interaction term between BMI and sex as well as stratification by sex were performed to investigate the varying extent of risk factors on diastolic function and LVMI in boys and girls. A *p*-value of 0.05 was considered statistically significant. Statistical analyses were performed using SAS 9.4 (Statistical Analysis Software, Cary, North Carolina, USA).

## Results

### Clinical Characteristics of the Study Population

Of the 356 children initially examined, 351 (187 boys; 53%) were included in further analysis. Four children were excluded due to preexisting heart conditions. Two-hundred-two were 2nd grade (8.21 ± 0.52 years of age) and 149 were 5th grade pupils (11.41 ± 0.55 years of age). Both sexes were equally represented in both groups. Anthropometrics, demographical characteristics and clinical details are shown in Table 1. One-hundred-two children (29%) had a BMIz ≥ 1.04 and 55 (16%) had a BMIz ≥ 1.64. The prevalence of overweight children was similar between 2nd and 5th graders (27% vs. 32%; *p* = 0.2814), while the prevalence of obesity tended to be lower in 2nd grade children (13% vs. 19%; *p* = 0.0987). The mean BMIz was elevated in both age groups (2nd grade: 0.28; 5th grade: 0.45). The mean waist-circumference *z*-score increased with age (0.69 vs. 0.91).

**Table 1:** Characteristics of the study population

Variables	Total			Second grade			Fifth grade			*p*
	MW	SD	*N*	MW	SD	*N*	MW	SD	*N*	
Age (years)	9.58	1.67	351	8.21	0.52	202	11.41	0.55	149	< 0.0001
Female sex	47% (164/351)			47% (95/202)			46% (69/149)			0.8481
Weight (kg)	36.83	12.34	351	30.21	6.87	202	45.80	12.45	149	< 0.0001
Height (cm)	139.82	11.55	351	132.04	6.45	202	150.36	8.03	149	< 0.0001
BMI (kg/m^2^)	18.40	3.85	351	17.19	2.97	202	20.04	4.29	149	< 0.0001
BMI *z*-score	0.36	1.12	351	0.28	1.09	202	0.45	1.15	149	0.1583
Overweight (BMI *z*-score ≥ 1.04)	29% (102/351)			27% (54/202)			32% (48/149)			0.2814
Obese (BMI *z*-score ≥ 1.64)	16% (55/351)			13% (26/202)			19% (29/149)			0.0987
Waist circumference (cm)	70.35	11.91	351	65.28	8.56	202	77.21	12.40	149	< 0.0001
Waist circumference *z*-score	0.78	0.84	351	0.69	0.84	202	0.91	0.83	149	0.0136
Systolic BP (mmHg)	104.04	8.21	351	102.07	7.67	202	106.71	8.20	149	< 0.0001
Systolic BP *z*-score	0.30	0.74	351	0.35	0.71	202	0.24	0.77	149	0.1653
Systolic BP *z*-score ≥ 1.282	35 (10%)			19 (9%)			16 (11%)			0.6805
Systolic BP *z*-score ≥ 1.64	12 (3%)			7 (3%)			5 (3%)			0.9554
Diastolic BP (mmHg)	60.80	5.64	351	59.60	5.35	202	62.42	5.62	149	< .0001
Diastolic BP *z*-score	0.05	0.49	351	0.06	0.47	202	0.04	0.52	149	0.7223
Diastolic BP *z*-score ≥ 1.282	5 (1%)			2 (1%)			3 (2%)			0.4239
Diastolic BP *z*-score ≥ 1.64	1 (0%)			1 (1%)			0 (0%)			0.9555
Physical fitness (W/kg)	3.07	0.68	337	3.09	0.69	196	3.04	0.66	141	0.5149
Cholesterol (mg/dl)	169.38	28.31	274	167.90	27.58	157	171.36	29.26	117	0.3177
HDL (mg/dl)	57.41	12.18	273	57.87	12.14	156	56.81	12.25	117	0.4804
LDL (mg/dl)	97.11	24.03	273	94.33	21.06	156	100.80	27.15	117	0.0335
Triglycerides (mg/dl)	81.48	44.93	274	75.05	41.16	157	90.10	48.39	117	0.0059
Biparental migration	47% (165/351)			46% (93/202)			48% (72/149)			0.6254

*BMI* body mass index, *BP* blood pressure, *HDL* high density lipoprotein, *LDL* low density lipoprotein

### Echocardiographic Findings of the Study Group

**Fig. 1:**
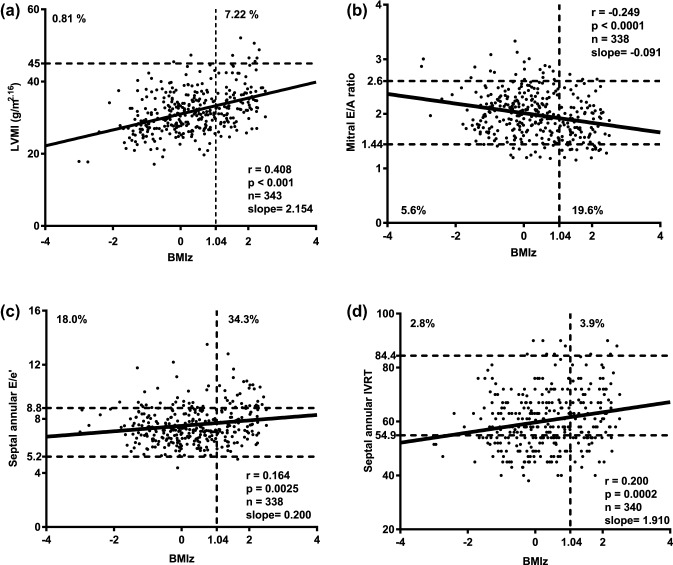
Echocardiographic parameters in conjunction with increasing BMIz. **a** Higher BMIz was associated with higher left ventricular mass/height ^2.16^. 7.22% of the overweight and obese children, but only 0.81% of those with BMIz < 1.04 had left ventricular hypertrophy (*p* < 0.001). The dotted line delineates 45 g/m^2.16^ as the upper normal limit for LVMI. **b** Using the current reference values from Eidem et al., 19.6% of the overweight or obese, but only 5.6% of the children with a BMIz < 1.04 had a mitral valve *E*/*A *ratio below the lower limit. **c** Using the current reference values from Eidem et al., 34.3% of the overweight or obese, but only 18% of the children with a BMIz < 1.04 had a septal annular *E*/*e*′ ratio above the upper limit. **d** Using the current reference values from Eidem et al., 3.9% of the overweight or obese, but only 2.8% of the children with a BMIz < 1.04 had an isovolumetric relaxation time (IVRT) above the upper limit. *BMIz* body mass index *z*-score, *E* peak early diastolic inflow Doppler velocity, *e* early diastolic annular myocardial velocity, *IVRT* isovolumetric relaxation time, *LVMI* left ventricular mass index, *TDI* tissue Doppler imaging

We present our data according to children’s BMIz (< 1.04 and ≥ 1.04) and in comparison to the current reference values published by Eidem et al. [11] (Table 2; Fig. 1). Statistically significant differences between children and adolescents with a BMIz ≥ 1.04 compared to those with BMIz below 1.04 were found for several structural and functional parameters. Left ventricular end-diastolic diameter *z*-score (LVEDdz) and LVMI [13] were significantly higher in overweight and obese children (*p* =  < 0.0001 and < 0.0001). Furthermore, several diastolic parameters were out of the normal range. While we cannot compare our values with those of the reference studies statistically [11], one can appreciate that for most values the exclusion of overweight and obese children resulted in more favorable values regarding function and morphology. According to the published reference data from Eidem et al., the number of out of range values are presented in Table 2. We only report those cases, in which the measurement pointed toward a potential unfavorable abnormality concerning diastolic function. The comparison of girls and boys revealed significant differences for the mitral and tricuspid *E*/*A *ratio as well as for the systolic PV flow (Supplementary Table S1).

**Table 2:** Echocardiographic Parameters compared to normal values from Eidem et al. [11]

Variables	7–9 Years					10–13 Years				
	BMI *z*-score < 1.04	Out of range^A^	BMI *z*-score ≥ 1.04	Out of range^A^	Eidem	BMI *z*-score < 1.04	Out of range^A^	BMI *z*-score ≥ 1.04	Out of range^A^	Eidem
	*N* = 143		*N* = 53		*N* = 55	*N* = 101		*N* = 46		*N* = 55
Weight (kg)*^#^	26.9 ± 3.6 (17.3–38.7)		39.2 ± 5.4 (28.1–53.7)		33.8 ± 14.9	39.2 ± 7.1 (24.1–58.6)		59.4 ± 10.4 (40.6–80.4)		47.2 ± 16.3
BSA (m^2^)*^#^	0.99 ± 0.09 (0.73–1.26)		1.21 ± 0.1 (0.99–1.49)		1.07 ± 0.27	1.27 ± 0.14 (0.93–1.66)		1.58 ± 0.17 (1.17–1.9)		1.37 ± 0.29
Age (years)*	8.2 ± 0.5 (7.1–9.8)		8.3 ± 0.5 (7.6–9.3)		7.91 ± 1.12	11.4 ± 0.6 (10.2–13.5)		11.4 ± 0.5 (10.6–12.4)		11.99 ± 1.11
Male	78 (53%)		29 (54%)		49%	57 (56%)		23 (48%)		69%
Heart rate (bpm)	78 ± 10 (56–105)		79 ± 11 (50–102)		80 ± 11	76 ± 11 (52–116)		79 ± 11 (59–109)		75 ± 12
**Left ventricle**	*N* = 142		*N* = 41		*N* = 55	*N* = 88		*N* = 32		*N* = 55
Mitral E velocity (cm/s)	102.8 ± 11.2 (76.2–133)	3 (2%)	104.3 ± 14.3 (75.6–139)	2 (4%)	94.4 ± 14.8	98 ± 13.8 (69–145)	9 (9%)	98.5 ± 16.1 (73.8–142)	5 (11%)	94.5 ± 16.0
Mitral A velocity (cm/s)*^#^	49.9 ± 9.3 (32.7–86.4)	14 (10%)	56.8 ± 11.9 (34.8–87)	16 (32%)	49.4 ± 12.5	51.5 ± 10.1 (32.4–78.6)	16 (16%)	57.4 ± 9.1 (43.8–81.3)	11 (24%)	49.5 ± 13.8
Mitral E/A ratio *^#^	2.1 ± 0.4 (1.2–3.3)	7 (5%)	1.9 ± 0.4 (1.2–2.7)	10 (20%)	2.0 ± 0.51	2.0 ± 0.4 (1.2–3.1)	7 (7%)	1.7 ± 0.3 (1.2–2.6)	10 (22%)	2.02 ± 0.58
IVRT (PW)*	52 ± 8.2 (36–76)		55.4 ± 6.8 (40–67)			57.3 ± 8.4 (40–76)		56.2 ± 7.2 (40–74)		
IVCT (PW)*	68.3 ± 12.4 (44–108)		64.1 ± 7.7 (49–84)			70.3 ± 10.1 (49–103)		69.8 ± 12.5 (49–105)		
**Tissue Doppler imaging**	*N* = 125		*N* = 49		*N* = 55	*N* = 97		*N* = 41		*N* = 55
Mitral annular *e*′-wave velocity*	20.3 ± 2.7 (12.9–27.4)	2 (1%)	19.3 ± 3 (13.3–27.1)	1 (2%)	17.2 ± 3.7	19.7 ± 2.8 (12.3–27.8)	10 (10%)	19 ± 3.1 (11.9–28.4)	11 (25%)	19.6 ± 3.4
Mitral annular *a*′-wave velocity*	6.5 ± 1.5 (4.1–13.4)	12 (9%)	7.5 ± 1.7 (4.2–12.1)	11 (21%)	6.7 ± 1.9	6.4 ± 1.5 (3.5–12.4)	8 (8%)	6.4 ± 1.5 (3–9.5)	7 (16%)	6.4 ± 1.8
Mitral annular *E*/*e*′*	5.1 ± 0.8 (3.4–7.2)	0 (0%)	5.5 ± 1 (3.2–7.5)	0 (0%)	5.8 ± 1.9	5 ± 0.9 (3.4–10.5)	8 (8%)	5.3 ± 1.2 (3.4–9.3)	7 (16%)	4.9 ± 1.3
Mitral annular IVRT	52.6 ± 8 (38–76.5)	1 (1%)	53.1 ± 7.2 (38–74)	0 (0%)	62.9 ± 11.9	57.5 ± 9.9 (38–85)	2 (2%)	55.4 ± 9.5 (40–72)	0 (0%)	62.6 ± 12.4
Septal annular *e*′-wave velocity*^#^	14.4 ± 1.8 (8.5–18.5)	10 (7%)	13.6 ± 1.7 (11–18.1)	3 (6%)	13.4 ± 1.9	13.3 ± 1.9 (8–18.3)	24 (24%)	12.1 ± 1.8 (7.4–17.3)	16 (36%)	14.5 ± 2.6
Septal annular *a*′-wave velocity*^#^	6.3 ± 1.2 (4.2–11.4)	35 (24%)	6.8 ± 1 (4.7–9.3)	18 (35%)	5.9 ± 1.3	5.8 ± 1.1 (3.6–10.1)	3 (3%)	6.3 ± 1 (4.3–8.9)	1 (2%)	6.1 ± 2.3
Septal annular *E*/*e*′*^#^	7.3 ± 1.1 (5.2–12.2)	13 (9%)	7.8 ± 1.3 (5.6–11.7)	12 (23%)	7.2 ± 1.6	7.5 ± 1.4 (4.4–13.5)	32 (32%)	8.3 ± 1.7 (5–12.8)	23 (51%)	6.6 ± 1.4
Septal annular IVRT*^#^	55.7 ± 8.7 (38–76)	0 (0%)	59.0 ± 8.9 (40–76)	0 (0%)	65.6 ± 10.7	64.2 ± 10.5 (45–90)	7 (7%)	68 ± 11.1 (45–90)	4 (9%)	72.5 ± 12.3
**Right ventricle**	*N* = 134		*N* = 46		*N* = 55	*N* = 95		*N* = 44		*N* = 55
Tricuspid E velocity (cm/s)	59.4 ± 7.7 (41.3–76.8)	7 (5%)	61 ± 8.7 (43.8–90)	1 (2%)	60.5 ± 13.9	61.1 ± 9.5 (39.4–90)	4 (4%)	59.8 ± 10.9 (36–100.5)	4 (9%)	59.6 ± 11.4
Tricuspid A velocity (cm/s)	38.4 ± 8 (21.4–67.2)	0 (0%)	40.4 ± 9 (24.5–60.6)	0 (0%)	42.4 ± 10.8	40 ± 9.4 (20.3–70)	0 (0%)	43.3 ± 9.1 (30–68)	0 (0%)	39.2 ± 11.3
Tricuspid E/A ratio^#^	1.61 ± 0.34 (0.88–2.67)	5 (4%)	1.57 ± 0.34 (1.07–2.83)	1 (2%)	1.49 ± 0.40	1.59 ± 0.36 (0.85–2.57)	5 (5%)	1.40 ± 0.26 (0.62–2.20)	6 (14%)	1.61 ± 0.47
**Tissue Doppler imaging**	*N* = 131		*N* = 44		*N* = 55	*N* = 92		*N* = 37		*N* = 55
Tricuspid annular *e*′-wave velocity*	16.2 ± 2.3 (10.6–23.3)	13 (9%)	14.8 ± 2.5 (10.5–20)	17 (34%)	16.5 ± 3.0	14.3 ± 3.2 (6–23.9)	40 (41%)	13.8 ± 2.5 (9–21)	14 (38%)	16.5 ± 3.1
Tricuspid annular *a*′-wave velocity	8.8 ± 2.2 (4.6–15.6)	0 (0%)	9.1 ± 2.3 (5.1–17.1)	0 (0%)	9.8 ± 2.7	8.6 ± 2.6 (4.4–17.5)	0 (0%)	9.2 ± 2.1 (6–16)	0 (0%)	10.3 ± 3.4
Tricuspid annular *E*/*e*′*	3.7 ± 0.7 (2.5–5.9)	0 (0%)	4.2 ± 0.7 (3–5.4)	0 (0%)	3.6 ± 0.8	4.5 ± 1.5 (2.8–13)	0 (0%)	4.4 ± 1 (2.6–7.3)	0 (0%)	3.5 ± 1.4
PV S-wave*	52.6 ± 8.1 (32.4–74.4)	0 (0%)	57.1 ± 8.9 (41.3–85.5)	0 (0%)	50.7 ± 11.3	49.8 ± 8.7 (32–79.4)	0 (0%)	51.4 ± 7.6 (35–72.5)	0 (0%)	49.0 ± 11.1
PV D-wave	67.7 ± 8.6 (34.8–87)	1 (1%)	66.6 ± 8.7 (42.8–81.6)	0 (0%)	53.3 ± 11.4	66.8 ± 9 (47.4–99)	0 (0%)	66.1 ± 5.9 (53.7–77.6)	0 (0%)	58.4 ± 12.1
EF Simpson biplane*	68 ± 2.9 (57.3–75.6)		66.6 ± 3.2 (57–73.2)			68.2 ± 3.5 (56.8–78)		69 ± 3.2 (62.8–76.5)		
**M-mode**	*N* = 134		*N* = 47			*N* = 95		*N* = 40		
IVSd*^#^	0.58 ± 0.07 (0.43–0.77)		0.64 ± 0.08 (0.51–0.88)			0.65 ± 0.09 (0.48–0.9)		0.72 ± 0.09 (0.57–0.9)		
LVEDd*^#^	3.86 ± 0.27 (3.11–4.74)		3.98 ± 0.28 (3.51–4.71)			4.14 ± 0.36 (3.38–5.08)		4.37 ± 0.39 (3.7–5.67)		
LVEDdz*^#^	0.01 ± 0.75 (− 2.31 to 2.19)		− 0.76 ± 0.68 (− 2.03 to 1.12)			− 0.48 ± 0.77 (− 2.14 to 1.78)		− 1.03 ± 0.91 (− 3.06 to 1.86)		
LVPWd*^#^	0.58 ± 0.07 (0.4–0.79)		0.63 ± 0.08 (0.44–0.81)			0.62 ± 0.09 (0.4–0.9)		0.7 ± 0.1 (0.56–1)		
LVMI (g/m^2.16^)*^#^	30.9 ± 5.2 (17.1–47.3)		34 ± 5.9 (24.7–52.1)			30 ± 5.7 (17.8–45.5)		35.9 ± 6.1 (25.8–50.6)		
LVMI (g/m^2.7^)*^#^	27 ± 4.6 (14.7–40.9)		29 ± 4.9 (21.2–43.3)			24.4 ± 4.4 (14.7–37.1)		28.7 ± 4.7 (20–40.2)		
LVMz*^#^	− 1.48 ± 0.95 (− 5.03 to 0.9)		− 0.98 ± 0.89 (− 2.7 to 1.27)			− 1.95 ± 1.15 (− 5.11 to 0.53)		− 0.95 ± 0.9 (− 3.18 to 0.84)		

*BSA* body surface area, *E* peak early mitral inflow Doppler velocities, *A* peak late mitral inflow Doppler velocities, *e′* early diastolic annular myocardial velocity, *a′* late diastolic annular myocardial velocity, *IVRT* isovolumetric relaxation time, *IVCT* isovolumetric contraction time, *PV S-wave* pulmonary venous flow velocity systolic, *PV D-wave* pulmonary venous flow velocity diastolic, *EF* ejection fraction, *IVSd* interventricular septal thickness end-diastolic, LVEDd left ventricular end-diastolic dimension, *LVEDdz* left ventricular end-diastolic dimension *z*-score, *LVPWd* left ventricular posterior wall dimension end-diastolic, *LVMI* left ventricular mass indexed for height^2.16^, *LVMz* left ventricular mass *z*-score adjusted for height

*Significant differences in the group of 7–9 year olds, ^#^significant differences in the group of 10–13 year olds

^A^Number of patients in which the measurement pointed toward a potential unfavorable abnormality (± 1 SD compared to Eidem et al.) concerning diastolic function

In the following, we will highlight particular morphological and functional aspects of the cohort.

### Left Ventricular Mass Index (LVMI)

**Fig. 2:**
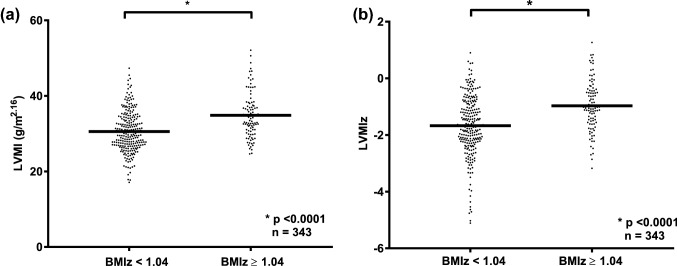
Left ventricular mass index (LVMI) and left ventricular mass index *z*-score (LVMIz) stratified according to children’s BMI *z*-score (BMIz). **a** Non-overweight children (BMIz < 1.04) had a significant lower LVMI than children overweight/obese (BMIz ≥ 1.04). **b** Children with BMIz < 1.04 had a significant lower LVMIz than children with a BMIz ≥ 1.04. *BMI* body mass index, *BMIz* body mass index *z*-score, *LVMI* left ventricular mass index, *LVMIz* left ventricular mass index *z*-score

As indicated above, higher BMI *z*-scores were associated with increases in LVMI (*p* < 0.001, Fig. 2). To explore which other factors contributed to a higher LVMI, we performed a multivariable linear regression analysis (Table 3). In addition to BMI, we found that sex, age and physical fitness were independent predictors of a higher LVMI. Importantly, we could not find an association between LVMI and systolic or diastolic BP in our cohort of healthy school children.

**Table 3:** Standardized models for the endpoints of different echocardiographic parameters

LVMI				LVMz			
	*β*	SD	*p*		*β*	SD	*p*
Intercept	32.466	0.8696	0.0007	Intercept	− 1.3483	0.1527	0.0126
Girls	− 21.0763	5.1787	< 0.0001	Girls	− 4.2042	0.9497	< 0.0001
Boys	0			Boys	0		
Age	− 19.8192	5.6944	0.0006	Age	− 5.9264	1.0495	< 0.0001
BMI	60.0535	7.1486	< 0.0001	BMI	10.9623	1.367	< 0.0001
Physical fitness	20.8346	6.9082	0.0028	Physical fitness	3.6986	1.2615	0.0036
				Systolic BP right arm	− 1.4964	1.0137	0.1409

Mitral *E*/*A* ratio				Mitral annular *E*/*e*′			
	*β*	SD	*p*		*β*	SD	*p*
Intercept	2.0144	0.04536	0.0005	Intercept	5.1167	0.105	0.0004
Girls	− 0.8167	0.3862	0.0352	Girls	− 1.1741	0.9041	0.195
Boys	0			Boys	0		
Age	− 0.6634	0.4343	0.1276	Age	− 2.0024	1.0048	0.0471
BMI	− 1.4953	0.4231	0.0005	BMI	3.199	0.9763	0.0012
Diastolic BP right arm	− 1.0966	0.4029	0.0069				

Septal annular *E*/*e*′				Septal annular IVRT			
	*β*	SD	*p*		*β*	SD	*p*
Intercept	7.6456	0.1711	0.0005	Intercept	60.5364	2.338	0.0015
Girls	− 1.6997	1.3082	0.1948	Girls	6.0434	9.2896	0.5158
Boys	0			Boys	0		
Age	0.786	1.4675	0.5926	Age	34.0376	10.5477	0.0014
BMI	5.4841	1.4157	0.0001	BMI	36.3556	9.9999	0.0003
				Diastolic BP right arm	29.1607	9.5348	0.0024
				Heart rate	− 46.9614	9.343	< .0001

Tricuspid *E*/*A* ratio				PV D-wave			
	*β*	SD	*p*		*β*	SD	*p*
Intercept	1.6628	0.09019	0.0029	Intercept	67.0637	0.4690	< 0.0001
Girls	− 0.5104	0.3263	0.1188	Girls	− 18.5681	8.8170	0.0360
Boys	0			Boys	0		
Age	− 0.2558	0.377	0.4979	Age	1.6547	9.3987	0.8604
BMI	− 0.9516	0.355	0.0077	BMI	− 20.6708	11.9846	0.0855
				Physical fitness	− 12.3673	11.5314	0.2843

*LVMI* left ventricular mass indexed for height^2.16^, *LVMz* left ventricular mass *z*-score adjusted for height, *E* peak early mitral inflow Doppler velocities, *A* peak late mitral inflow Doppler velocities, *e*′ early diastolic annular myocardial velocity, *IVRT* isovolumetric relaxation time, *PV D-wave* pulmonary venous flow velocity diastolic

**Fig. 3:**
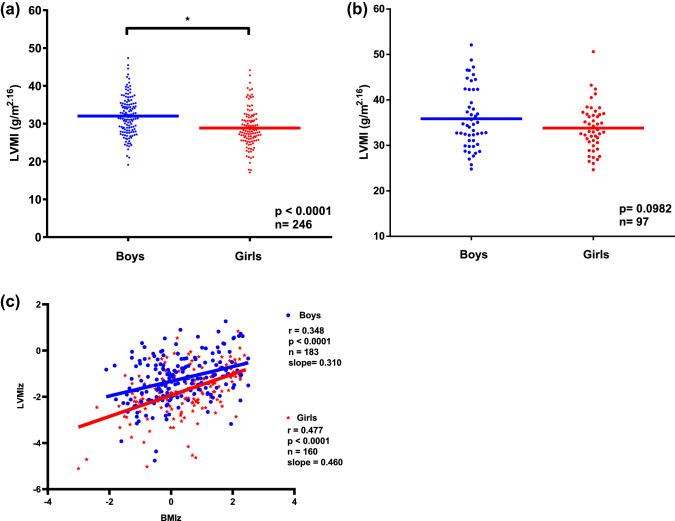
Left ventricular mass demonstrated for boys and girls separated according to children’s BMIz. **a** Significant difference in LVMI between boys and girls with a BMIz < 1.04. **b** The difference of LVMI between boys and girls was no longer found in children with a BMIz ≥ 1.04. **c** There is a sex-specific difference between the slopes for LVMI with increasing BMIz. *BMIz* body mass index *z*-score, *LVMI* left ventricular mass index

We further explored sex differences and found higher LVMI in boys than in girls for children with a BMIz < 1.04 (*p* < 0.0001), but this difference was no longer observed in overweight and obese children (*p* = 0.096, Fig. 3). We performed a sex stratified analysis to investigate how changes in BMI affect LVMI in each sex (Table 4). Our results support the observation that girls and boys with higher BMI values have comparable absolute LVMI values. We show that, e.g., at a BMI value of 30 kg/m^2^ girls are expected to have an estimated LVMI of 37.9 g/m^2^ whereas boys will have 39.5 g/m^2^. This convergence at higher BMI values is the result of a greater increase in LVMI for a given BMI change. Per 1 kg/m^2^ BMI increase LVMI increases by 0.5272 g/m^2.16^ in boys, whereas by 0.7336 g/m^2.16^ in girls.

**Table 4:** Interaction terms between sex and BMI as well as stratification by sex

	LVMI				LVMz		
	*β*	SD	*p*		*β*	SD	*p*
Intercept	27.7298	2.4435	0.0077	Intercept	− 1.1683	0.4406	0.1177
Age	− 0.544	0.1867	0.0038	Age	− 0.1854	0.03379	< 0.0001
Girls	− 6.3765	2.7264	0.0199	Girls	− 1.2689	0.4941	0.0107
Boys	0			Boys	0		
BMI	0.6236	0.1076	< 0.0001	BMI	0.1014	0.0195	< 0.0001
BMI * Girls	0.1794	0.1456	0.2187	BMI * Girls	0.03799	0.02638	0.1508
BMI * Boys	0			BMI * Boys	0		
Intercept	24.5598	2.3497	0.009	Intercept	− 2.3085	0.4236	0.0321
BMI * Boys	0.5272	0.1109	< 0.0001	BMI * Boys	0.07035	0.01923	0.0003
Intercept	16.8644	1.9312	0.0129	Intercept	− 3.8709	0.4164	0.0114
BMI * Girls	0.7336	0.09871	< 0.0001	BMI * Girls	0.1173	0.02042	< 0.0001

	Septal annular IVRT				PV D-wave		
	*β*	SD	*p*		*β*	SD	*p*
Intercept	41.8147	4.8547	0.0132	Intercept	66.6673	3.6558	0.003
Age	1.5155	0.3518	< 0.0001	Age	− 0.04501	0.2947	0.8787
Girls	− 10.6783	5.0561	0.0354	Girls	8.1222	4.4779	0.0706
Boys	0			Boys	0		
BMI	0.2529	0.1997	0.2064	BMI	0.09589	0.1739	0.5817
BMI * Girls	0.5525	0.2698	0.0414	BMI * Girls	− 0.54	0.2378	0.0238
BMI * Boys	0			BMI * Boys	0		
Intercept	52.5448	5.2389	0.0098	Intercept	66.3696	3.0802	0.0021
BMI * Boys	0.4507	0.2031	0.0278	BMI * Boys	0.08843	0.1662	0.5953
Intercept	40.884	4.3985	0.0114	Intercept	74.5022	3.2425	0.0019
BMI * Girls	1.0609	0.1989	< 0.0001	BMI * Girls	− 0.4517	0.1697	0.0086

*LVMI* left ventricular mass indexed for height^2.16^, *LVMz* left ventricular mass *z*-score adjusted for height, *IVRT* isovolumetric relaxation time, *PV D-wave* pulmonary venous flow velocity diastolic

We also calculated LVMIz [20], expressing LV mass relative to LBM, meant as sensitivity analysis. Similar to our calculations based on LVMI by Chinali et al., we only saw very few children with LV hypertrophy. Using LBM normalized values we did not expect to see an effect of BMI. However, we confirmed our previous observation that higher BMI values were associated with smaller increases in LVM in boys (boys: *β* =  − 0.033, *p* = 0.045; girls: *β* =  − 0.005, *p* = 0.72) (Supplementary Table S2).

### Left Ventricular Diastolic Function

As indicated above overweight and obese children also tended to have more unfavorable echocardiographic diastolic parameters than children with BMIz < 1.04. Early mitral and tricuspid inflow velocities (*E*) were similar between the groups, but both mitral and tricuspid A-wave velocities were increased, resulting in a decreased *E*/*A* ratio. The values for the *E*/*e*′ ratio (septal and mitral annular) were higher in overweight and obese children and the IVRT measured at the septal annulus was significant longer. The effect of increasing BMIz is also highlighted in Fig. 1.

Multivariable linear regression analysis was used to further explore contributing factors for the selected diastolic parameters (Table 3). The *E*/*A *ratio of the mitral valve was independently associated with sex, BMI and diastolic BP. Girls showed a significant lower *E*/*A *ratio compared to boys while age had no effect. Interestingly higher BMI was associated with an increase in the A-wave, while the E-wave essentially was left unchanged. *E*/*e*′ ratio (either septal or mitral) were only predicted by BMI without a contribution of sex and age. IVRT was independently associated with age, BMI and diastolic BP, while heart rate was inversely related.

**Fig. 4:**
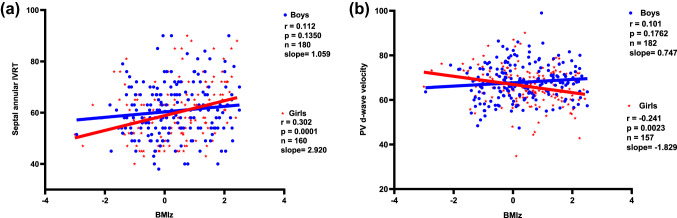
**a** Sex-specific difference between the slopes for a isovolumetric relaxation time (IVRT) with increasing BMIz and **b** for diastolic pulmonary venous flow (PV d-wave) with increasing BMIz. *BMIz* body mass index *z*-score, *IVRT* isovolumetric relaxation time, *PVd* pulmonary venous flow velocity diastolic

We assumed again an interaction with sex. By creating an interaction term (Table 4; Fig. 4a), we demonstrated a significantly higher increase for IVRT in girls for a given BMI. Sex stratified regression models showed how BMI affect IVRT in boys and girls differently. We found a prolonged IVRT of 0.5525 ms for a given BMI in girls compared to boys. The stratified regression analysis demonstrated that a BMI increase of 1 kg/m^2^ was associated with an IVRT increase of 0.4507 ms in boys (*p* = 0.028), while in girls IVRT extended significantly more with 1.0609 ms (*p* < 0.001).

Interestingly this phenomenon was also found in the association between reduction of diastolic PV flow and BMI, in which girls showed a reduction of 0.54 m/s with a BMI increase of 1 kg/m^2^ compared to boys. The sex stratified regression model confirmed a reduced diastolic PV flow with increasing BMI in girls (Fig. 4b; Table 4).

## Discussion

This cross-sectional study on echocardiographic changes in a large cohort of apparently healthy German school children significantly extends the available data currently used to assess morphological and functional cardiac aspects. We provide values for many parameters used in daily clinical assessment for two age groups separated by children’s BMI. We show significant structural (LVMI) and functional (A-wave velocities, *E*/*A *ratio, pulmonary flow velocities) differences between girls and boys. Our data confirm the importance of overweight and obesity for many of the parameters used in routine work-up of patients. We extend current data by showing a greater effect of overweight and obesity on different echocardiographic parameters in girls.

### Sex Differences

Sex differences in LVMI as an important structural parameter have been repeatedly reported [22]. However, information about sex-related differences in diastolic echocardiographic parameters in children is scarce most likely due to the limited number of cases available in earlier reference studies [23, 24]. Values for several echocardiographic parameters measured in girls from our study were found in the respective upper or lower ranges of normality, indicating a real biological difference between the sexes. Apart from those physiological differences, we demonstrate that in girls the same BMI increase resulted in a greater increase in LVMI as well as a greater increase in IVRT and diastolic PV flow compared to boys. These findings suggest that there are sex-related negative effects of being overweight or obese on mass as well as compliance of the left ventricle with increasing BMI. We therefore assume a higher susceptibility toward cardiac changes in presence of increasing BMI as an important cardiovascular risk factor in girls compared to boys. Our findings in these otherwise healthy but overweight children are in accordance with data from a large cohort of children with chronic kidney disease, in whom a stronger association of obesity with LVMIz and LVH was demonstrated among girls compared to boys [7]. A greater vulnerability of girls upon cardiovascular stresses had been demonstrated in older girls after renal transplantation that displayed higher BP values when exposed to an additional risk, i.e., higher trough levels for cyclosporine A [25]. Further support comes from recent adult data. The protective effect of female sex on CV health is lost in presence of additional comorbidities and stresses (such as type 2 diabetes, hypertension, hypercholesterinaemia, sedentary lifestyle, mental stress) [26–28].

While peripubertal and perimenopausal differences point toward a hormonal cause for CVD risk [29], our data suggest that risk factors earlier in life must be taken into account, as most of our study population had not reached puberty yet. A possible explanation for our findings is a significantly higher adipose mass in females than in males for each BMI category, as described by De Simone et al. [30] and for pediatric age by Taylor et al. [31]. Adipose tissue is considered to be a metabolic highly active tissue and a large endocrine organ [32] expressing several hormones, growth factors and cytokines [33, 34]. It has been shown that especially in women adiposity and inflammation pathways are highly relevant in the development of CVD [35]. Lau et al. [36] demonstrated sex differences in circulating biomarkers, with significant higher levels of leptin and ceruloplasmin in women than in men. For leptin levels, there were found associations with diastolic function (*E*/*e*′) in adults, after adjusting for age, sex and BMI [37]. Higher ceruloplasmin levels were associated with heart failure and were weakly associated with CVD [38]. Unfortunately we do not have information on leptin, ceruloplasmin or other factors derived from adipose tissue of our cohort. Notably, a correlation of high-sensitive C-reactive protein with echocardiographic parameters was not found.

### Impact of Obesity

The importance of obesity for the different echocardiographic parameters has been controversially discussed especially concerning echocardiographic parameters of diastolic function. But in nearly all studies, indices of LV mass were greater in overweight and obese than in normal weight children or adolescents [39–42]. It should be noted that, the prevalence of left ventricular hypertrophy in overweight children varies dependent of the method used for normalization [43]. Children in our study showed a higher LVMI and LVMz with increasing BMI, which is in agreement with previous pediatric studies, describing a correlation between BMI and LVMI [4, 5, 39, 44–47]. In agreement with recent results from larger cohorts of children [41] the influence of BP, often discussed as possible reason for a higher LVMI in overweight children [4], could not be confirmed in our cohort.

In adulthood early subclinical changes of diastolic parameters are very sensitive indicators for disturbances of myocardial energy metabolism [48]. It is known that, overweight and obesity lead to energetic abnormalities and insufficient energy supply of the myocardium [49], but the evaluation of diastolic function is still a very complex field of science. Our data on lower LV compliance in case of overweight and obesity resemble data from larger studies [5]. Di Salvo et al. investigated 150 obese children and adolescents and showed a significant higher *E*/*e*′ ratio as well as a longer IVRT in these subjects in comparison to a control group [50]. Dhuper et al. presented a higher *E*/*e*′ ratio and lower mitral *E*/*A *ratio in 213 obese children, while subjects with hypertension were not excluded and the effect of obesity solely could not be rated [46]. While some smaller studies could not show any differences between overweight and normal weight [44], others did but with heterogeneous results for the different parameters describing diastolic function [4, 5, 40]. Especially a difference in *E*/*e*′ and *E*/*A *ratio could be observed in several studies [4, 39, 46, 51]. A possible explanation for these partly contradicting results may be that in presence of chronic volume overload—as assumed in overweight and obesity—only minimal changes in tissue Doppler velocities can be expected [52]. Studies with rather smaller sample sizes might have had not enough power to show significant differences in all the parameters.

Our findings suggest that changes in cardiac structure start early in life and occur at much lower BMIz level than expected. This implicates an urgent need for an effective obesity prevention in children and adolescents, with a special focus on obesity-related cardiac alterations in girls.

### Study Limitations

Potential limitation of the current study is the lack of information about the pubertal status of our study participants. Since sex-specific hormone levels play a significant role for cardiovascular health in adults, a closer investigation of pubertal status would have been favorable. This cross-sectional study design does not provide information about the potential progression of the described cardiac alterations in adulthood or potential reversibility. So the clinical significance of these findings remains unknown and will require longitudinal follow-up over several years to determine their predictive value. LVMI and left ventricular diastolic function were probably also influenced by other factors that could not be examined, e.g. hyperinsulinemia or leptin levels.

## Conclusion

There are significant structural and functional sex differences in a number of echocardiographic parameters in children and adolescents. Our data confirm the importance of overweight and obesity for many of the parameters used in routine work-up of patients with changes in myocardial structure and function, indicating an early onset of potentially unfavorable alterations in the myocardium. We demonstrate a higher susceptibility of girls by showing a negative effect of being overweight or obese on different echocardiographic parameters in girls. Further studies are needed to explore how the knowledge on sex-specific risk factors can be implemented in current risk management strategies to maximize benefit especially for girls.

## Supplementary Information

Below is the link to the electronic supplementary material.

Supplementary file1 (DOCX 25 kb)

## Data Availability

Data available upon reasonable request to the authors.

## Abbreviations

BMI: Body mass index
BMIz: Body mass index *z*-score
BP: Blood pressure
BSA: Body surface area
CVD: Cardiovascular disease
HDL: High-density lipoprotein cholesterol
LDL: Low-density lipoprotein cholesterol
*A*: Peak late mitral inflow Doppler velocities
*a*′: Late diastolic annular myocardial velocity
*E*: Peak early mitral inflow Doppler velocities
*e*′: Early diastolic annular myocardial velocity
EF: Ejection fraction
IVCT: Isovolumetric contraction time
IVRT: Isovolumetric relaxation time
IVSd: Interventricular end-diastolic septum thickness
LVEDd: Left ventricular end-diastolic dimension
LVEDdz: Left ventricular end-diastolic dimension *z*-score
LVM: Left ventricular mass
LVMz: Left ventricular mass *z*-score
LVMI: Left ventricular mass index
LVMIz: Left ventricular mass index *z*-score
LVPWd: Left ventricular end-diastolic posterior wall thickness
PV D-wave: Diastolic pulmonary venous flow velocity
PV S-wave: Systolic pulmonary venous flow velocity

## Code Availability

Not applicable.
