# Supplementary material for: Impact of Sex and Obesity on Echocardiographic Parameters in Children and Adolescents
Source: Pediatr Cardiol. 2022 Apr 8;43(7):1502–16. doi: 10.1007/s00246-022-02876-2 (PMC9489563; doi:10.1007/s00246-022-02876-2)
Supplement: Supplementary file 1 — Supplementary file1 (DOCX 25 kb) [file 246_2022_2876_MOESM1_ESM.docx]

**Supplement**

Journal: Pediatric Cardiology, Springer

# **Impact of Sex and Obesity on Echocardiographic Parameters in Children and Adolescents**

Jeannine von der Born^1*^, MD, Sarah Baberowski^1*^, Nima Memaran^1^, MD, Lena Grams^2^, Denise Homeyer^2^, MA, Bianca Borchert-Mörlins^3^, MD, Rizky Indrameikha Sugianto^1^, DDS, PhD, Mira Paulsen^1^, Elena Bauer^1^, BSc, Grabitz, Carl^1^, MD, Bernhard M.W. Schmidt^5^, MD MSc, Arno Kerling^2^, MD, Philipp Beerbaum^6^, MD, Meike Stiesch^4^, DMD, Uwe Tegtbur^2^, MD, Anette Melk^1^, MD, PhD

*equal contribution

1 Department of Pediatric Kidney, Liver and Metabolic Diseases, Hannover Medical School, Hannover, Germany;

2 Institute of Sports Medicine, Hannover Medical School, Hannover, Germany

3 Department of Cardiology and Angiology, Hannover Medical School, Hannover, Germany

4 Department of Prosthetic Dentistry and Biomedical Material Research, Hannover Medical School, Hannover, Germany

5 Department of Nephrology and Hypertension, Hannover Medical School, Hannover, Germany

6 Department of Pediatric Cardiology and Pediatric Intensive Care Medicine

**Corresponding author:**

Anette Melk, MD, PhD

[melk.anette@mh-hannover.de](mailto:melk.anette@mh-hannover.de) ; ORCID: 0000-0002-8164-6318

Department of Pediatric Kidney, Liver and Metabolic Diseases,

Hannover Medical School, Carl-Neuberg-Str. 1, 30625 Hannover, Germany

| **Table S1** Echocardiographic Parameters of the study population separated by sex | | | | | | | | | |  |
| --- | --- | --- | --- | --- | --- | --- | --- | --- | --- | --- |
|  | | | | | | | | | |  |
|  |  |  | **Boys** |  | **Girls** | |  | | | |
| **Variables** |  |  | **N=183** |  | **N=160** | | **p** | | | |
| Weight (kg) |  |  | 36.3 ± 11.7 (17.3-80.4) |  | 37.4 ± 13.1 (20.2-78.9) | | n.s. | | | |
| BSA (m^2^) |  |  | 1.18 ± 0.22 (0.74-1.84) |  | 1.19 ± 0.24 (0.82-1.79) | | n.s. | | | |
| Age (Years) |  |  | 9.6 ± 1.7 (7.5-12.8) |  | 9.5 ± 1.6 (7.1-13.5) | | n.s. | | | |
| Heart rate (bpm) |  |  | 76 ± 10 (50-116) |  | 80 ± 10 (55-104) | | 0.0003 | | | |
| **Left ventricle** |  |  | **N= 148** |  | **N= 132** | |  | | | |
| Mitral E velocity (cm/s) |  |  | 101.8 ± 14 (69-142) |  | 100.2 ± 12.7 (74.4-145) | | n.s. | | | |
| Mitral A velocity (cm/s) |  |  | 51.3 ± 9.6 (32.4-86.4) |  | 53.7 ± 11 (32.7-87) | | 0.0349 | | | |
| Mitral E/A ratio |  |  | 2 ± 0.4 (1.2-3.3) |  | 1.9 ± 0.4 (1.2-3.1) | | 0.0260 | | | |
| IVRT (PW) |  |  | 54.8 ± 8.4 (36-76) |  | 54.5 ± 8.1 (37-76) | | n.s. | | | |
| IVCT (PW) |  |  | 68.6 ± 12.1 (47-108) |  | 68.3 ± 10.3 (44-103) | | n.s. | | | |
| **Tissue Doppler imaging** |  |  | **N= 162** |  | **N=150** | |  | | | |
| Mitral annular e´-wave velocity |  |  | 19.8 ± 3 (11.9-27.8) |  | 19.8 ± 2.7 (13-28.4) | | n.s. | | | |
| Mitral annular a´-wave velocity |  |  | 6.6 ± 1.5 (3-12.1) |  | 6.6 ± 1.7 (3.5-13.4) | | n.s. | | | |
| Mitral annular E/e´ |  |  | 5.2 ± 1 (3.4-10.5) |  | 5.1 ± 0.8 (3.2-7.9) | | n.s. | | | |
| Mitral annular IVRT |  |  | 55.2 ± 9.2 (40-85) |  | 53.9 ± 8.6 (38-74) | | n.s. | | | |
| Septal annular e´-wave velocity |  |  | 13.6 ± 1.9 (8-18.5) |  | 13.7 ± 2 (7.4-18.5) | | n.s. | | | |
| Septal annular a´-wave velocity |  |  | 6.1 ± 1.2 (3.6-11.4) |  | 6.4 ± 1.1 (3.8-10.1) | | n.s. | | | |
| Septal annular E/e´ |  |  | 7.6 ± 1.4 (4.4-13.5) |  | 7.5 ± 1.3 (5-12.8) | | n.s. | | | |
| Septal annular IVRT |  |  | 60.6 ± 10.6 (38-90) |  | 60.1 ± 10.7 (40-90) | | n.s. | | | |
| **Right ventricle** |  |  | **N=166** |  | **N=153** | |  | | | |
| Tricuspid E velocity (cm/s) |  |  | 59.9 ± 8.9 (36-100.5) |  | 60.6 ± 8.9 (41.3-90) | | n.s. | | | |
| Tricuspid A velocity (cm/s) |  |  | 38.9 ± 8.8 (20.3-70) |  | 40.9 ± 8.9 (21.4-68) | | 0.0464 | | | |
| Tricuspid E/A ratio |  |  | 1.6 ± 0.4 (0.9-2.8) |  | 1.5 ± 0.3 (0.6-2.5) | | 0.0450 | | | |
| **Tissue Doppler imaging** |  |  | **N=159** |  | **N=148** | |  | | | |
| Tricuspid annular e´-wave velocity |  |  | 15 ± 2.9 (6-23.9) |  | 15.2 ± 2.6 (9-22.1) | | n.s. | | | |
| Tricuspid annular a´-wave velocity |  |  | 8.4 ± 2.1 (4.4-16) |  | 9.4 ± 2.5 (5.1-17.5) | | 0.0002 | | | |
| Tricuspid annular E/e´ |  |  | 4.2 ± 1.3 (2.5-13) |  | 4.1 ± 0.8 (2.6-7.3) | | n.s. | | | |
| PV S-wave |  |  | 50.5 ± 8.5 (32-79.4) |  | 54.5 ± 8.3 (32.4-85.5) | | <0.0001 | | | |
| PV D-wave |  |  | 67.9 ± 8.3 (47.4-99) |  | 66.1 ± 8.4 (34.8-90.1) | | n.s. | | | |
| EF Simpson biplane |  |  | 67.8 ± 3.2 (60.7-76.4) |  | 68.1 ± 3.2 (56.8-78) | | n.s. | | | |
| **M-Mode** |  |  | **N= 172** |  | **N= 147** | |  | | | |
| IVSd |  |  | 0.64 ± 0.09 (0.45-0.9) |  | 0.61 ± 0.09 (0.43-0.9) | | 0.0035 | | | |
| LVEDd |  |  | 4.06 ± 0.37 (3.19-5.67) |  | 3.99 ± 0.36 (3.11-4.9) | | n.s. | | | |
| LVEDdz |  |  | -0.25 ± 0.92 (-3.06-2.19) |  | -0.54 ± 0.75 (-2.55-2.07) | | 0,0018 | | | |
| LVPWd |  |  | 0.63 ± 0.09 (0.45-0.9) |  | 0.6 ± 0.1 (0.4-1) | | 0.0045 | | | |
| LVMI (g/m^2.16^) |  |  | 33.1 ± 5.9 (19.1-52.1) |  | 30.3 ± 5.6 (17.1-50.6) | | <0.0001 | | | |
| LVMI (g/m^2.7^) |  |  | 27.8 ± 5 (15.6-43.3) |  | 25.5 ± 4.5 (14.7-39.7) | | <0.0001 | | | |
| LVMz |  |  | -1.2 ± 1 (-4.8-1.3) |  | -1.7 ± 1.1 (-5.1-0.8) | | <0.0001 | | | |
| Aorta |  |  | 2.2 ± 0.2 (1.6-2.8) |  | 2.1 ± 0.2 (1.7-2.8) | | 0.0298 | | | |
| Left atrium |  |  | 2.8 ± 0.4 (2-4.1) |  | 2.8 ± 0.4 (2-3.8) | | n.s. | | | |
|  |  |  |  | |  |  | |  |  |  |
| *BSA* body surface area, *E* peak early mitral inflow Doppler velocities, *A* peak late mitral inflow Doppler velocities, *e’* early diastolic annular myocardial velocity,  *a’* late diastolic annular myocardial velocity, *IVRT* isovolumic relaxation time, *IVCT* isovolumic contraction time, *PVs* pulmonary venous flow velocity systolic, *PVd* pulmonary venous flow velocity diastolic, *EF* ejection fraction, *IVSd* interventricular septal thickness end-diastolic , LVEDd left ventricular end-diastolic dimension , *LVEDdz* left ventricular end-diastolic dimension z-score, *LVPWd* left ventricular posterior wall dimension end-diastolic , *LVMI* left ventricular mass indexed for height 2.16,  *LVMz* left ventricular mass z-score adjusted for height | | | | | | | | | |  |

| **Table S2:**  Interaction terms between sex and BMI as well as stratification by sex | | | |
| --- | --- | --- | --- |
|  | | | |
| **LVMIz (LBM)** | | | |
|  | **β** | **SD** | **p** |
| **Intercept** | 1.041 | 0.3648 | 0.104 |
| **Age** | -0.0859 | 0.02789 | 0.0022 |
| **Girls** | -0.5256 | 0.4072 | 0.1977 |
| **Boys** | 0 | . | . |
| **BMI** | -0.01769 | 0.01607 | 0.2716 |
| **BMI*Girls** | 0.02385 | 0.02174 | 0.2734 |
| **BMI*Boys** | 0 | . | . |
|  |  |  |  |
| **Intercept** | 0.5422 | 0.349 | 0.2605 |
| **BMI*Boys** | -0.03284 | 0.01629 | 0.0453 |
|  |  |  |  |
| **Intercept** | -0.1976 | 0.2855 | 0.5604 |
| **BMI*Girls** | -0.00541 | 0.01494 | 0.7175 |
|  |  |  |  |
| *LVMIz (LBM)* Left ventricular mass index z-score normalized for LBM | | | |
